# Blood Leukocyte Signaling Pathways as Predictors of Severity of Acute Pancreatitis

**DOI:** 10.1097/MPA.0000000000001832

**Published:** 2021-05-20

**Authors:** Antti Turunen, Antti Kuuliala, Harri Mustonen, Pauli Puolakkainen, Leena Kylänpää, Krista Kuuliala

**Affiliations:** From the ∗ Abdominal Center, Department of Abdominal Surgery; † Bacteriology and Immunology, Helsinki University Hospital and University of Helsinki, Helsinki, Finland.

**Keywords:** pancreatitis, prognosis, blood, leukocytes, signal transduction, flow cytometry, AF - Alexa Fluor, AP - acute pancreatitis, AUC - areas under the ROC curves, CT - computed tomography, FITC - fluorescein isothiocyanate, HC - healthy control, HLA-DR - human leukocyte antigen-DR, IL – interleukin, LPS - lipopolysaccharide, MMS - Modified Marshal Score, NF-κB - nuclear fact-κB, OD - organ dysfunction, p - phosphorylation levels, PE - phycoerythrin, PerCP - peridinin chlorophyll protein complex, RA - rheumatoid arthritis, ROC - receiver operating characteristic, Ser - serine, STAT - signal transducer and activator of transcription, Tyr - tyrosine

## Abstract

Clinical practice lacks biomarkers to predict the severity of acute pancreatitis (AP). We studied if intracellular signaling of circulating leukocytes could predict persistent organ dysfunction (OD) and secondary infections in AP.

**Methods:**

A venous blood sample was taken from 174 patients with AP 72 hours or less from onset of symptoms and 31 healthy controls. Phosphorylation levels (p) of appropriately stimulated signal transducer and activator of transcription 1 (STAT1), STAT6, nuclear factor-κB (NF-κB), Akt, and nonstimulated STAT3 in monocytes, neutrophils, and lymphocytes was measured using phosphospecific flow cytometry.

**Results:**

The patients showed higher pSTAT3 and lower pSTAT1, pSTAT6, pNF-κB, and pAkt than healthy controls. pSTAT3 in all leukocyte subtypes studied increased, and pSTAT1 in monocytes and T cells decreased in an AP severity-wise manner. In patients without OD at sampling, high pSTAT3 in monocytes and T lymphocytes were associated with development of persistent OD. In patients with OD, low interleukin-4–stimulated pSTAT6 in monocytes and neutrophils and *Escherichia coli–*stimulated pNF-κB in neutrophils predicted OD persistence. High pSTAT3 in monocytes, CD8^+^ T cells, and neutrophils; low pSTAT1 in monocytes and T cells; and low pNF-κB in lymphocytes predicted secondary infections.

**Conclusions:**

Leukocyte STAT3, STAT1, STAT6, and NF-κΒ phosphorylations are potential predictors of AP severity.

Acute pancreatitis (AP) is a common disease with a global incidence of 33.7/100,000.^1^ In the Revised Atlanta Classification, AP is classified as mild, moderately severe, or severe.^2^ Patients with severe AP have persistent (>48 hours) organ dysfunction (OD) and much higher mortality and morbidity compared with mild or moderately severe AP.^3^ The course of AP comprises an initial hyperinflammatory phase and a later compensatory anti-inflammatory phase. During the latter, risk of secondary infections increases, having a major impact on mortality.^4^ However, the biphasic model is simplified, and actually complex individually varying immune system alterations occur over the disease course.^5,6^ A remarkable problem in clinical practice is the lack of tools to determine the immune-inflammatory status of each patient with AP and predict outcome at hospital admission.

Leukocyte signaling via, for example, nuclear factor-κB (NF-κB) and signal transducer and activator of transcription 3 (STAT3) pathways, plays a key role in systemic inflammation in AP.^7,8^ Notably, representing the early phase of leukocyte activation, preceding, for example, cytokine production, the signaling pathways provide attractive candidates for monitoring the development of AP. Furthermore, activation of these pathways can be rapidly measured, for example, by phosphospecific whole-blood flow cytometry. We have previously observed, using this method, several phosphorylation aberrations of NF-κB and STATs in different leukocyte subtypes of patients with severe AP, compared with healthy controls (HCs),^9–11^ and that these aberrations in sepsis and severe AP differ from those of HCs or patients with mild AP.^12^ Our findings on another systemic inflammatory disease, rheumatoid arthritis (RA), also suggest that STAT3 phosphorylation in circulating leukocytes is associated with disease activity,^13^ likewise the phosphorylation of Akt kinase^14^ implicated in anti-inflammatory responses.^15,16^ Human trials on Akt activity in AP are lacking.

We previously suggested that STAT3 and STAT1 phosphorylation in circulating leukocytes may predict AP severity.^12^ In the present larger prospective study, we determined NF-κB, STAT1, STAT3, STAT6, and Akt phosphorylation in blood leukocytes in relation to AP severity and potential to predict persistent OD and secondary infections.

## MATERIALS AND METHODS

**FIGURE 1:**
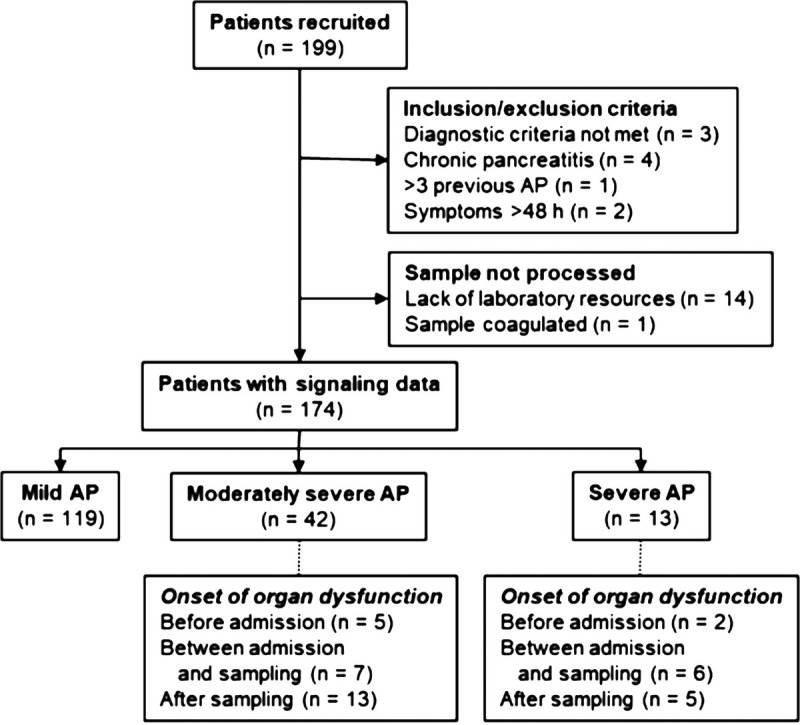
Flowchart of patient recruitment and outcome.

Of the 199 patients with AP enrolled at the emergency department at Helsinki University Hospital between December 2013 and May 2019, 174 were included in the final study (Fig. 1). Thirty-one healthy volunteers served as controls (HC; mean age, 45 years; 57% women). The study was approved by the Surgical Ethical Review Board (Joint Authority of the Helsinki and Uusimaa Hospital District). Written informed consent was obtained from each patient or next of kin.

The Revised Atlanta Classification was used to diagnose and classify AP.^2^ Diagnosis warranted abdominal pain typical of AP and elevation of 3 times or more the upper limit of serum amylase level and/or typical finding in computed tomography. Acute pancreatitis was classified as mild (no local or systemic complication and no OD), moderately severe (local complications, aggravation of chronic illness or transient OD <48 hours), or severe (persistent OD ≥48 hours). The Modified Marshall Score (MMS) was used to diagnose OD.^17^ A score of 2 or higher in respiratory, cardiovascular, or renal system was diagnostic for OD. Necrotizing AP was detected with contrast-enhanced computed tomography.

Occurrence of secondary infections was determined from patient records. Because each case of cholecystitis was diagnosed concomitantly with AP, it was not included in secondary infections.

Exclusion criteria were age less than 18 years, chronic pancreatitis, time from onset of symptoms longer than 48 hours, known malignancy, or more than 2 previous AP attacks.

### Blood Sampling

An 8-mL venous blood sample was taken 24 hours or less from admission into a polypropylene tube (Becton Dickinson [BD], Lincoln Park, NJ) supplemented with 1:10 pyrogen-free citrate phosphate dextrose (Baxter Health Care Ltd, Norfolk, UK). The samples were kept at room temperature and stimulated within 3 hours.

### Biological Agents and Leukocyte Agonists

Fluorescein isothiocyanate (FITC)–conjugated anti-CD14 [clone MφP9, immunoglobulin G (IgG)_2b_], phycoerythrin (PE)–conjugated anti–human leukocyte antigen (HLA)–DR (clone L243, IgG_2a_) with isotype control (mouse IgG_2a_), peridinin chlorophyll protein complex (PerCP)–conjugated anti-CD3 (clone SK7, IgG_1_), PE-CF594–conjugated anti-CD4 (clone SK3, IgG_1_), and Alexa Fluor (AF)647- or PE-conjugated anti-CD8 (clone RPA-T8, IgG_1_) were used for surface marker staining. Phosphospecific labeling was done using AF647-conjugated anti-phosphorylated (p)STAT1 (pY701) (clone 4a, IgG_2a_), PE-conjugated anti-pSTAT3 (pY705) (clone 4/P-STAT3, IgG_2a_), AF647-conjugated anti-pSTAT3 (pS727) (clone 49/p-STAT3, IgG_1_), AF647-conjugated anti-pSTAT6 (pTyr641) (clone 18/p-STAT6, IgG_2a_), AF647-conjugated anti-pNF-κB p65 (pS529) (clone K10–895.12.50, IgG_2b_), and PE-conjugated anti-pAkt (pSer473) (clone M89–61, IgG_1_). All antibodies were from BD Biosciences (San Jose, Calif).

Interleukin (IL)-4 and IL-6 were from R&D Systems (Minneapolis, Minn). *Escherichia coli* O111:B4 lipopolysaccharide (LPS) was from Sigma (St. Louis, Mo). Whole *E. coli* bacteria were from the Finnish Institute for Health and Welfare (Helsinki, Finland), stored in glycerol-tryptone soy broth medium at −70°C, washed, and pelleted. All agonists were diluted in phosphate-buffered saline before use.

### Ex Vivo Stimulations and Immunolabeling

One hundred–microliter blood aliquots were pipetted into polypropylene tubes (Beckman Coulter, Brea, Calif) so that for each stimulation there was an unstimulated control tube. The stimulations were as follows: IL-6 (100 ng/mL, 5 minutes; tubes 1 and 2), IL-4 (10 ng/mL, 10 minutes; 3 and 4), *E. coli* (50 cells/leukocyte, 10 minutes; 5 and 6), or LPS (100 ng/mL, 10 minutes; 7 and 8). In addition, 2 unstimulated aliquots were prepared for constitutive pSTAT3 determination (tubes 9 and 10). The cell surface marker antibodies compatible with the subsequent permeabilization procedure were added so that the total incubation time was 15 minutes: all aliquots were supplemented with anti–CD14-FITC (5 μL), tubes 1, 2, 9, and 10 with anti–CD4-PE-CF594 (3 μL), and tube 9 [for pSTAT3(pTyr705)] with anti–CD8-AF647 (4 μL).

Leukocyte fixation, erythrocyte lysis, and leukocyte permeabilization after stimulations were done according to BD Phosflow Protocol III for Human Whole Blood.^18^ Next, the samples were washed with and resuspended in BD Pharmingen Stain Buffer.

Tubes 1 and 2 were supplemented with anti–pSTAT1-AF647 (5 μL), anti–CD8-PE (5 μL), and anti–CD3-PerCP (8 μL), tubes 3 and 4 with anti–pSTAT6-AF647 (5 μL), tubes 5 and 6 with anti–pNF-κB-AF647 (5 μL), and tubes 9 and 10 with anti–pAkt-PE (5 μL). Tube 10 for pSTAT3(pSer727) measurement was supplemented with anti–pSTAT3(pSer727)-AF647 (5 μL), anti–CD8-PE (5 μL), and anti–CD3-PerCP (8 μL), and tube 9 with anti–pSTAT3(pTyr705)-PE (5 μL) and anti–CD3-PerCP (8 μL). The samples were incubated in the dark at room temperature for 20 minutes, washed, resuspended in 300 μL of Stain Buffer, and stored on ice in the dark until flow cytometric run is performed within 3 hours.

### Flow Cytometry

Cyan Advanced Digital Processing flow cytometer (Beckman Coulter) was used for data acquisition. Data were analyzed using FlowJo 10.6 software (Tree Star, Ashland, Ore).

Monocytes were identified using CD14-FITC positivity and forward scatter and side scatter characteristics. Polymorphonuclear cells and lymphocytes were identified based on forward scatter and side scatter characteristics. T lymphocytes were identified using CD3-PerCP positivity and gated as CD4-PE-CF594 and CD8-PE or CD8-AF647 positive.

For each phosphorylation, corresponding PE or AF647 histograms were developed, and the mean fluorescence intensities were recorded in stimulated and nonstimulated samples. The stimulation-induced phosphorylation was calculated as the ratio of the mean fluorescence intensity of stimulated and nonstimulated samples. For constitutive STAT3 phosphorylation, the anti-pSTAT3–stained samples were compared with fluorescence minus one samples, and the ratio was calculated correspondingly.

The proportion of HLA-DR–positive monocytes was determined as described previously.^19^

### Statistical Analysis

We used IBM SPSS Statistics version 25 (IBM, Armonk, NY). Data are shown as means with ranges or medians with interquartile ranges. Phosphorylation levels were compared between HCs and disease severity–grouped patients with AP using Kruskal-Wallis test with post hoc Mann-Whitney test. Jonckheere-Terpstra test was used to test whether the phosphorylations associated with AP severity. Receiver operating characteristic curve analysis with respective areas under the receiver operating characteristic curves was obtained to compare each phosphorylation with respect to predicting severe AP or infection. In the analyses, *P* values less than 0.05 were considered statistically significant, double-sided tests were used, and no adjustment was made for multiple testing.

## RESULTS

Basic clinical features are shown in Table 1.

**TABLE 1:** Characteristics of the Patients

	Severity of AP		
	Mild (n = 119; 68%)	Moderately Severe (n = 42; 24%)	Severe (n = 13; 8%)
Sex, male, n (%)	82 (69)	28 (67)	11 (85)
Age, y	51.3 (20–89)	53.3 (24–85)	49.3 (25–81)
Etiology of AP, n (%)			
Alcohol	64 (54)	25 (60)	9 (69)
Biliary	27 (23)	8 (19)	2 (15)
Post-ERCP	1 (1)	3 (7)	0 (0)
Idiopathic	19 (16)	6 (14)	1 (8)
Other	8 (7)	0 (0)	1 (8)
Hospital length of stay, d	4.1 (1–15)	10.1 (2–37)	40.4 (6–94)
ICU on admission, n (%)	0 (0)	5 (12)	13 (100)
ICU length of stay, d	NA	4.4 (3–6)	19.6 (3–55)
Plasma C-reactive protein on admission, mg/L	36.1 (3–252)	29.9 (3–261)	59.7 (3–466)
Plasma C-reactive protein at sampling, mg/L	81.2 (3–400)	92.5 (3–308)	149.7 (5–495)
Plasma creatinine on admission, μmol/L	70.5 (32–144)	74.5 (32–172)	109.2 (64–295)
Plasma creatinine at sampling, μmol/L	64.1 (32–128)	66.2 (31–158)	134.9 (52–292)
Monocyte HLA-DR expression at sampling, %	82 (16–99)	73 (31–96)	65 (22–95)
30-Day mortality, n (%)	0 (0)	0 (0)	5 (39)
SOFA score on admission	0.9 (0–4)	1.8 (0–7)	3.3 (0–9)
SOFA score at sampling	1.1 (0–5)	2.3 (0–6)	4.5 (1–9)
APACHE II score on admission	5.3 (0–12)	6.5 (0–16)	7.9 (3–15)
APACHE II score at sampling	5.1 (0–13)	7.1 (3–15)	9.3 (1–15)
MMS on admission	0.2 (0–1)	0.5 (0–2)	0.7 (0–3)
MMS at sampling	0.4 (0–1)	0.9 (0–3)	1.8 (0–5)
Time from onset to admission, h	19.9 (1–48)	18.7 (2–48)	21.8 (3–48)
Time from onset to sampling, h	36.3 (7–71)	33.9 (9–74)	34.9 (9–71)
First AP, n (%)	87 (73)	29 (69)	11 (85)
Local complication, n (%)	0 (0)	26 (62)	10 (77)
Pancreatic necrosis, n (%)	0 (0)	13 (31)	11 (85)
CT, n (%)	60 (50)	35 (83)	12 (92)
MRI, n (%)	30 (25)	9 (21)	0 (0)
ERCP, n (%)	9 (8)	2 (5)	3 (23)
Necrosectomy, n (%)	0 (0)	1 (2)	5 (39)
Open abdomen, n (%)	0 (0)	0 (0)	3 (23)
Secondary infection, n (%)			
Pneumonia	1 (14)	5 (46)	1 (9)
Urinary tract infection	1 (14)	3 (27)	0 (0)
Cholecystitis	3 (43)	1 (9)	0 (0)
Intra-abdominal infection	0 (0)	0 (0)	9 (82)
Other	2 (29)	2 (18)	1 (9)
Bacteremia	1 (1)	2 (5)	4 (31)

Data are shown as mean (range) or number of patients (percentage).

APACHE II indicates Acute Physiology and Chronic Health Evaluation II; CT, computed tomography; ERCP, endoscopic retrograde cholangiopancreatography; ICU, intensive care unit; MRI, magnetic resonance imaging; NA, not applicable; SOFA, sequential organ failure assessment.

### Phosphorylations of Patients With Mild, Moderately Severe, and Severe AP and HC

**FIGURE 2:**
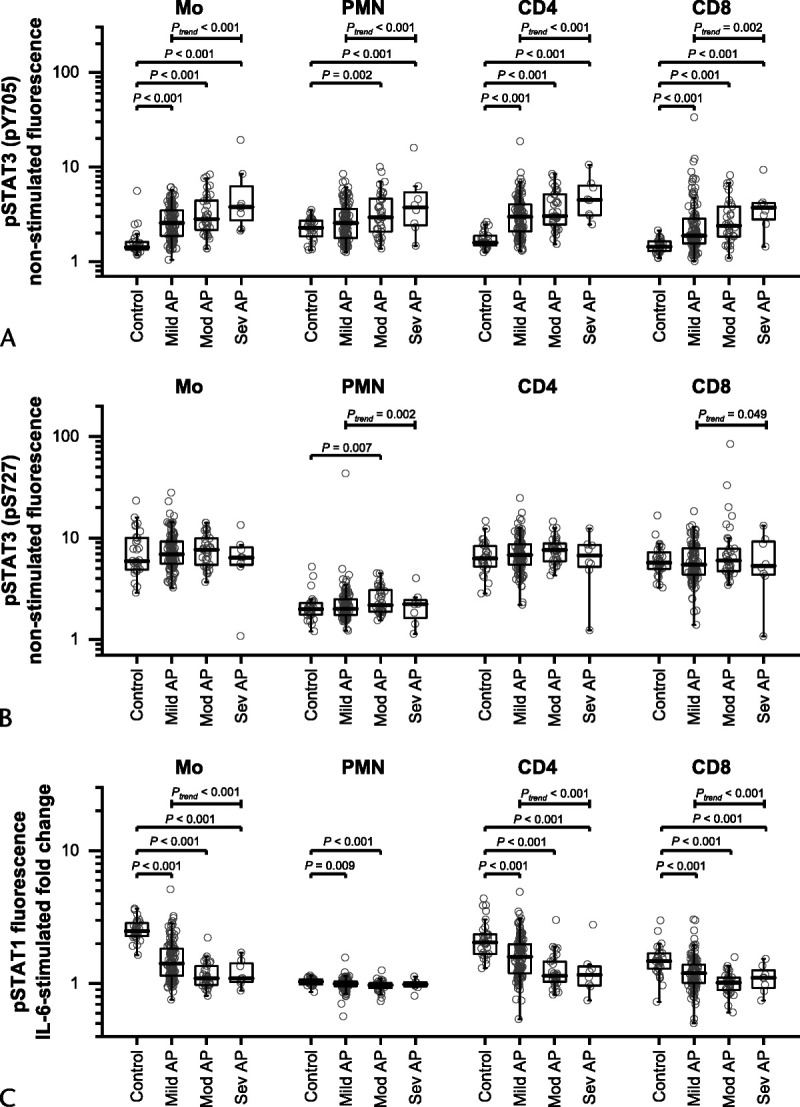
Nonstimulated pSTAT3 fluorescence (A, pY705; B, pS727) and IL-6–stimulated pSTAT1 fluorescence (C) in monocytes (Mo), polymorphonuclear cells (PMN), CD4^+^ T lymphocytes (CD4), and CD8^+^ T lymphocytes (CD8) of HCs (Control) and patients with mild (Mild AP), moderately severe (Mod AP), and severe AP (Sev AP). The nonstimulated fluorescence intensities are shown as ratios of mean fluorescence intensities of phosphospecific antibody-stained samples and unstained samples (fluorescence minus one), and the IL-6 stimulation is shown as ratio of mean fluorescence intensity of IL-6–stimulated and nonstimulated samples. Individual subjects are shown as gray circles, with the overlaid boxes representing group medians with 25th and 75th percentiles. The groups were compared with Kruskal-Wallis test, and the *P* values from post hoc Mann-Whitney tests comparing the HCs to patient groups are shown. The association with severity of AP was tested using Jonckheere-Terpstra test (*P*_trend_).

Constitutive tyrosine (Tyr)705 phosphorylation levels (p) of STAT3 were significantly higher in patients with AP than in HC in all leukocyte subtypes studied (Fig. 2A). There was a monotone increasing trend of pTyr705 level with increasing severity of AP. The constitutive serine p(Ser)727 levels of STAT3 generally did not differ between patients and HC, although a trend was observed in neutrophils and CD8^+^ T cells in relation to AP severity (Fig. 2B).

Interleukin-6–stimulated pSTAT1 levels in monocytes and CD4^+^ and CD8^+^ T cells were significantly lower in all AP groups than in HC, and the pSTAT1 levels showed a monotone decreasing trend in relation to AP severity (Fig. 2C).

Neutrophils are unresponsive to IL-6 because they lack the signal transducing component gp130.^20^ Therefore, the IL-6–stimulated neutrophil phosphorylation results were excluded from the analyses.

**FIGURE 3:**
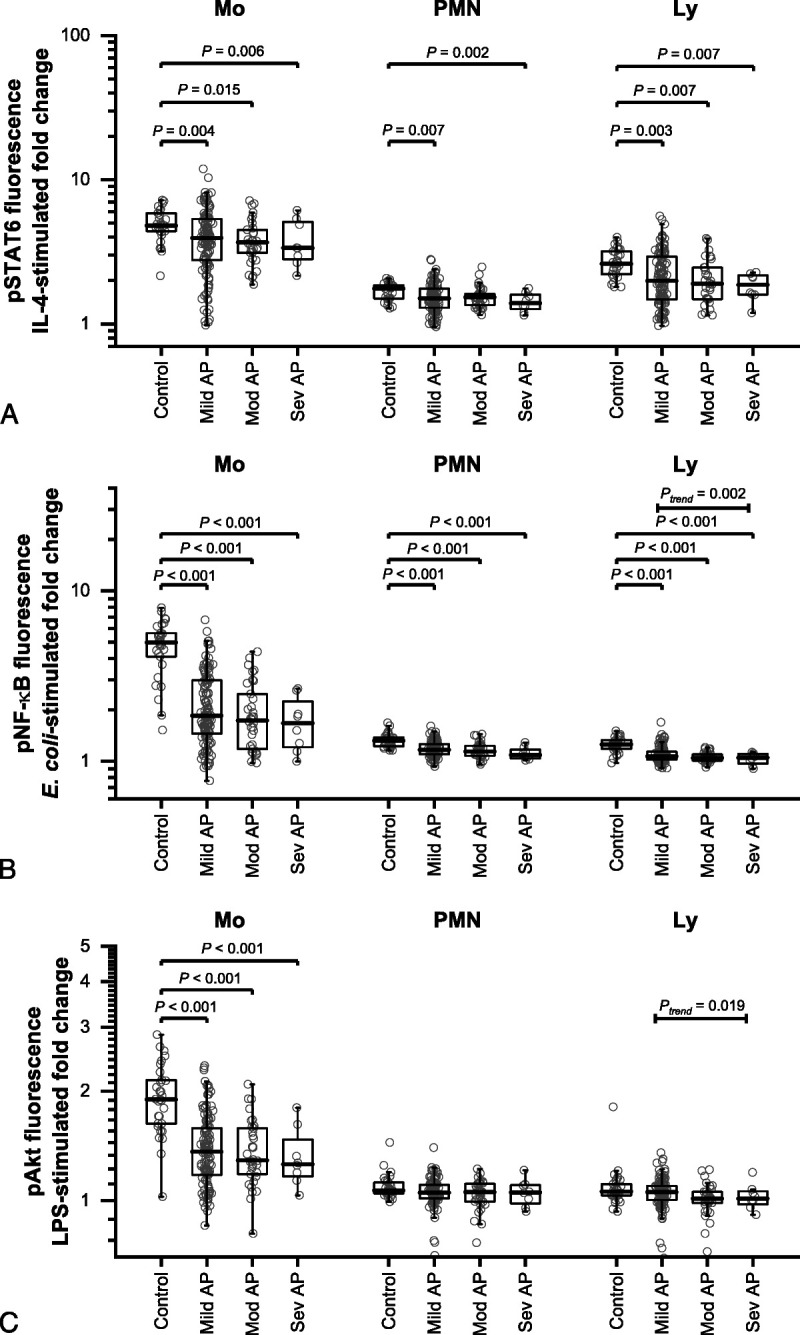
Interleukin-4–stimulated pSTAT6 (A), *E. coli*–stimulated pNF-κB (B), and LPS-stimulated pAkt fluorescence (C) in monocytes (Mo), polymorphonuclear cells (PMN), and lymphocytes (Ly) of HCs (Control) and patients with mild (Mild AP), moderately severe (Mod AP), and severe AP (Sev AP). The results are shown as ratio of mean fluorescence intensity of stimulated and nonstimulated samples. Individual subjects are shown as gray circles, with the overlaid boxes representing group medians with 25th and 75th percentiles. The groups were compared with Kruskal-Wallis test, and the *P* values from post hoc Mann-Whitney tests comparing the HCs to patient groups are shown. The association with severity of AP was tested using Jonckheere-Terpstra test (*P*_trend_).

Interleukin-4–stimulated pSTAT6 levels were lower in leukocytes of patients with AP compared with HC. The levels showed no trend in relation to AP severity (Fig. 3A).

The patient groups showed lower *E. coli*–stimulated pNF-κB levels compared with HC in all leukocyte subtypes studied. In lymphocytes, the levels showed a monotone decreasing trend in relation to AP severity (Fig. 3B).

Lipopolysaccharide-stimulated Akt phosphorylation was lower in all AP groups compared with HC in monocytes. The levels showed a monotone decreasing trend in relation to AP severity (Fig. 3C).

### Phosphorylations in Predicting Course of AP

#### Severe AP in Patients Without OD (MMS <2) at Sampling

**FIGURE 4:**
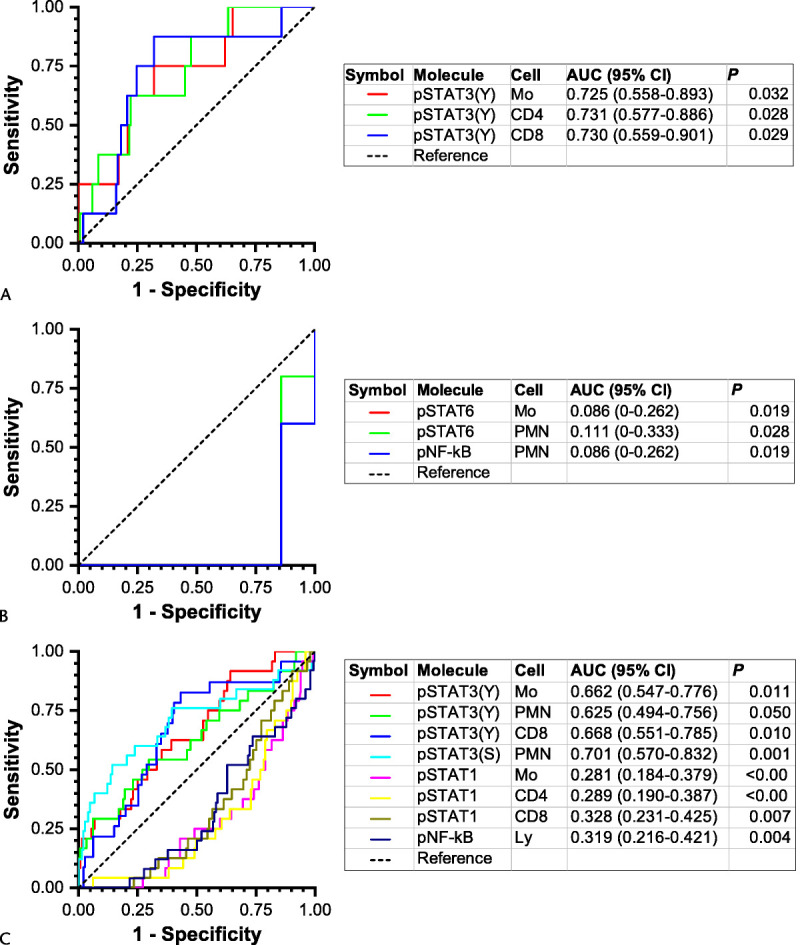
The receiver operating characteristic (ROC) curves and corresponding areas under curve (AUC) with 95% confidence intervals (CI) of (A) monocyte (Mo), CD4^+^ T lymphocyte (CD4), and CD8^+^ T lymphocyte (CD8) nonstimulated pSTAT3 (pY705) fluorescence in prediction of severe AP among patients without OD at study entry; (B) Mo and polymorphonuclear cell (PMN) IL-4–stimulated pSTAT6 fluorescence and PMN *E. coli*–stimulated pNF-κB fluorescence in prediction of severe AP among patients with OD at study entry; and (C) Mo, PMN, and CD8 nonstimulated pSTAT3 (pY705) fluorescence, PMN nonstimulated pSTAT3 (pS727) fluorescence, Mo, CD4, and CD8 IL-6–stimulated pSTAT1 fluorescence, and lymphocyte (Ly) *E. coli*–stimulated pNF-κB fluorescence in the prediction of secondary infections among all patients with AP. Note that in (B), the ROC curve shown in blue runs an identical course with the one shown in red, which is thus obscured.

Five patients developed severe AP with persistent OD after sampling. High constitutive STAT3 pTyr705 levels in monocytes and CD4^+^ and CD8^+^ lymphocytes predicted severe AP (Fig. 4A).

#### Severe AP in Patients With OD (MMS ≥2) at Sampling

Thirteen patients had MMS of 2 or higher at sampling, 6 of which subsequently developed severe AP. Low levels of IL-4–stimulated pSTAT6 in monocytes and neutrophils and *E. coli–*stimulated pNF-κB in neutrophils predicted severe AP (Fig. 4B).

#### Secondary Infections

The infections diagnosed during the hospitalization are shown in Table 1. Subsequent development of secondary infection was associated with high constitutive levels of STAT3 pTyr705 in monocytes, CD8^+^ T cells, and neutrophils, and those of pSer727 in neutrophils, and low levels of IL-6–stimulated pSTAT1 in monocytes and CD4^+^ and CD8^+^ T cells, and of *E. coli*-stimulated pNF-κB in lymphocytes (Fig. 4C).

## DISCUSSION

The results showed that in patients with AP, constitutive Tyr705 phosphorylation (pTyr705) levels of STAT3 in circulating leukocytes are associated with both disease severity and subsequent development of persistent OD, as determined by phosphospecific whole-blood flow cytometry. All the stimulation-induced phosphorylation levels studied, that is, those of pSTAT1, pSTAT6, pNF-κB, and pAkt, were lower in patients with AP than in HC in monocytes, commonly also in lymphocytes and neutrophils. In patients with AP with OD, the IL-4–stimulated pSTAT6 levels in monocytes and neutrophils and *E. coli*–stimulated pNF-κB levels in neutrophils were inversely associated with persistent nature of OD. The development of secondary infections was associated with STAT3 pTyr705 in monocytes, CD8^+^ T cells, and neutrophils, and with STAT3 pSer727 in neutrophils, and inversely with stimulated levels of pSTAT1 in monocytes and CD4^+^ and CD8^+^ T cells, and of pNF-κB in lymphocytes. To the best of our knowledge, these findings are novel.

The finding that constitutive STAT3 pTyr705 level in leukocytes can serve as a predictive marker of OD is supported by murine models of acute lung injury. In mice, small-molecule STAT3 inhibitor treatment suppressed lung inflammation.^21^ In alveolar macrophages, STAT3 activation, for example, via IL-33, leads to matrix metalloproteinase production and degradative processes. Neutralization of IL-33 alleviated acute lung injury in rats.^22^ Furthermore, as observed in mice, postseptic CD4^+^ T cells may contribute to secondary lung infection via STAT3-mediated IL-17 production.^23^ Altogether, the results suggest that determining and targeting STAT3 phosphorylation could be an advantageous approach in preventing OD and secondary infections in AP.

Constitutive STAT3 pTyr705 in leukocytes may result from elevated levels of circulating inflammatory STAT3-activating cytokines. These include IL-35 and IL-33, both implicated in the pathogenesis and severity of AP,^24,25^ and the “traditional” proinflammatory IL-6. We also found that in neutrophils, STAT3 pSer727 levels associated with development of secondary infections. However, with regard to the pSer727 levels even in severe AP remarkably overlapping with those of HC, this phosphorylation and the cytokines promoting it may only have a minor contribution to the course of AP.

It has been reported previously that acute alcohol intake activates STAT1/3 pathways and concomitantly induces the STAT3-inhibiting suppressors of cytokine signaling in monocytes, leading to down-regulation of IL-6–induced STAT1/STAT3 signaling.^26^ This seems compatible with our finding that IL-6–stimulated pSTAT1 levels of monocytes and T cells of patients with AP are low. However, alcohol intake may not explain our findings, because, first, the aberrant phosphorylation also concerned patients with nonalcohol etiology. Second, constitutive STAT1 phosphorylation was not observed in the patients' leukocytes. Third, we have previously found constitutive STAT3 phosphorylation in circulating leukocytes of patients with RA,^13^ suggesting an inflammatory origin for the finding. However, the possibility remains that individual differences in alcohol-induced leukocyte STAT3 phosphorylation may affect the susceptibility to or severity of AP.

According to our present findings, low NF-κB phosphorylation in response to *E. coli* encounter in neutrophils is associated with persistence of OD, and in lymphocytes with development of secondary infections in AP. As NF-κB is usually seen as a proinflammatory transcription factor, low capacity to phosphorylate it could refer to the presence of immunosuppression that follows the initial hyperinflammatory phase in AP. Mechanistically, it can comprise down-regulation of signaling proteins downstream of Toll-like receptors or by endogenous alarmins.^27,28^ In T cells, *E. coli* induces up-regulation of proinflammatory cytokines and down-regulation of the anti-inflammatory IL-10.^29^ Impaired NF-κB phosphorylation capacity may change this cytokine pattern with concomitant susceptibility to secondary infections. Noteworthy, among the numerous effects of NF-κB, there are also anti-inflammatory mechanisms, for example, negative regulation of the proinflammatory IL-1β secretion.^30^ It seems plausible that impaired NF-κB phosphorylation in leukocytes upon inflammatory stimulation can favor several mechanisms driving tissue injury and OD.

We also found that low IL-6–induced STAT1 phosphorylation, or IL-6 hyporesponsiveness, in circulating monocytes and CD8^+^ T cells is associated with the development of secondary infections in AP. This is rational with regard to the nonredundant functions of STAT1 in the defense against pathogens. Humans not expressing STAT1 die prematurely, and mice with STAT1 gene deletion die of severe infections soon after birth.^31^ In the present study, IL-6 hyporesponsiveness also associated with AP severity in monocytes and CD4^+^ T cells, further suggesting its suppressive effect on immune responses. The mechanism of IL-6 hyporesponsiveness can be IL-6R down-regulation as observed in monocytes of healthy subjects after interaction with bacterial antigens.^32^ Alternatively, IL-6 hyporesponsiveness may be due to attenuated gp130 phosphorylation as described in murine sepsis.^33^ If the latter is true in human AP, the hyporesponsiveness may be a broader phenomenon involving responses to other cytokine signaling via gp130 (eg, IL-11 and IL-27).^34^

Furthermore, the association between low IL-4-stimulated pSTAT6 levels in monocytes and neutrophils and persistence of OD in AP is likely to reflect the anti-inflammatory potential of the IL-4/STAT6 signaling pathway. As removal of IL-4 has been observed to cause STAT6 dephosphorylation in several cell types,^35^ cytokine imbalance in the hyperinflammatory phase of AP may reduce the ability to phosphorylate STAT6 in response to IL-4. It has also been reported that during inflammation, IL-4-receptor-α is up-regulated specifically on myeloid effector cells, thus priming them for STAT6 signaling, which can be a homeostatic mechanism to limit excessive inflammation and tissue damage.^36^ Whether this priming, or IL-4 signaling downstream, is disturbed in AP warrants further studies.

In murine models of sepsis/endotoxemia, Akt kinase has been implicated in several inflammation-limiting functions, for example, suppression of the production of inflammatory cytokines and coagulation, accompanied by significant improvement in survival.^15,16^ Recently, we found that LPS-induced Akt phosphorylation in monocytes is decreased in newly diagnosed untreated RA and even more in treatment-irresponsive chronic RA.^14^ In the present study, LPS-induced pAkt levels were lower in monocytes of patients with AP than in those of HC. In lymphocytes, the levels decreased along with the severity of AP but did not have predictive value. With regard to the LPS not being an ideal stimulus for lymphocytes, the possibility remains that other stimuli could reveal predictive potential for pAkt in AP.

The method we used, phosphospecific whole-blood flow cytometry, has several advantages that make it useful for studying clinical samples. It is minimally invasive, the amount of blood needed is small, the assay can be performed in hours, several targets can be detected in the same cells, and risk of contamination is small. In addition, immune cell activation via intracellular signaling pathways represents an early stage of systemic inflammatory response. In fact, based on our study,^9^ the method has been considered applicable to immune status determination before administering immune-modulatory therapies in inflammatory diseases.^37^ However, the assay is beyond routine laboratory testing, but instead, its implementation in clinical practice requires integrated translational medicine and standardization between distinct flow cytometers. In addition, further studies are needed to elucidate whether there are still more accurate predictive markers along the signaling pathways than the ones we found in the present study.

We showed that using phosphospecific whole-blood flow cytometry, it is possible to predict development of OD and secondary infections early in the course of AP. The predictive biomarkers were the levels of phosphorylated STAT3, STAT1, STAT6, and NF-κB. Further studies are warranted to elucidate if these biomarkers, determined using this rapid and minimally invasive method, can be used in tailoring personalized medicine and developing targeted drugs for patients with AP.
